# Safety and efficacy of interrupting dual antiplatelet therapy one month following percutaneous coronary intervention: a meta-analysis of randomized controlled trials

**DOI:** 10.1186/s12872-022-02900-6

**Published:** 2022-10-28

**Authors:** Shane Parfrey, Amr Abdelrahman, Daniel Blackman, Jonathan M. Blaxill, Michael S. Cunnington, John P. Greenwood, Christopher J. Malkin, Abdul M. Mozid, Jennifer A. Rossington, Murugapathy Veerasamy, Nancy Wassef, Stephen B. Wheatcroft, Heerajnarain Bulluck

**Affiliations:** 1 Yorkshire Heart Centre, Leeds General Infirmary, Leeds Teaching Hospitals NHS Trust, Great George Street, Leeds, LS1 3EX UK; 2 Leeds Institute of Cardiovascular and Metabolic Medicine, University of Leeds, Leeds, UK

**Keywords:** Dual antiplatelet therapy, Percutaneous coronary intervention, one-month, Major bleeding, Mortality, Myocardial infarction, Stent thrombosis, Stroke, Randomized controlled trials

## Abstract

Very short duration of dual antiplatelet therapy (DAPT) following percutaneous coronary intervention (PCI) has recently attracted a lot of attention with the introduction of newer generations stents. This is appealing, especially in patients at high bleeding risk. However, none of the trials were powered for the individual ischemic and bleeding endpoints. All randomised controlled trials (RCTs) investigating one-month versus routine duration of DAPT in patients undergoing PCI and reporting outcomes from the time of cessation of DAPT (1 month) to 1 year were eligible for inclusion in the meta-analysis. The pooled risk ratios (RR) with their 95% confidence interval (CI) were calculated with the random-effects model using the Mantel-Haenszel method. Four RCTs involving 26,576 patients were included in this meta-analysis. Cessation of DAPT after 1 month was associated with significantly less major bleeding [RR 0.70, 95%CI (0.51–0.95), *P* = 0.02, heterogeneity (I^2^) = 42%]. There was no statistically significant difference in all-cause mortality [RR 0.84 (95%CI 0.69–1.03), *P* = 0.10, I^2^ = 0%] and stroke [RR 0.71 (95%CI 0.45–1.13), *P* = 0.15, I^2^ = 42%] when compared to routine duration of DAPT. There was also no difference in myocardial infarction (MI) [RR 1.12 (95%CI 0.91–1.39), *P* = 0.28, I^2^ = 0%], and definite or probable stent thrombosis [RR 1.49 (95%CI 0.92–2.41), *P* = 0.11, I^2^ = 0%] with cessation of DAPT after 1 month. Cessation of DAPT 1 month after PCI was associated with significantly less major bleeding, but there was no difference in the rate of all-cause mortality, stroke, MI and stent thrombosis.

**Supplementary Information:**

The online version contains supplementary material available at 10.1186/s12872-022-02900-6.

## Introduction

Dual antiplatelet therapy (DAPT) with aspirin and a P2Y12 inhibitor is recommended in patients undergoing percutaneous coronary intervention (PCI) and has been shown to reduce ischemic complications [1, 2]. Prolonged treatment with DAPT is associated with a higher bleeding risk. However, the incidence of late and very late stent thromboses have reduced significantly with the emergence of newer generation drug-eluting stent (DES) [3] and the latter is also preferred over bare metal stents in high bleeding risk (HBR) patients [4, 5]. Therefore, very short duration of DAPT following PCI has recently attracted a lot of attention. The MASTER DAPT (Dual Antiplatelet Therapy after PCI in Patients at High Bleeding Risk) trial [6] was recently published and presented at the recent European Society of Cardiology (ESC) 2021 Meeting. It showed that among HBR patients undergoing PCI, cessation of DAPT after 1 month was noninferior to the continuation of therapy for at least 2 additional months with regards to the occurrence of net adverse clinical events [6]. One-month DAPT also resulted in a lower incidence of major bleeding or clinically relevant non-major bleeding [6]. Although this is the largest trial to date investigating one-month DAPT in HBR patients and included 4434 patients, it was not powered to look at the individual components of the composite endpoint such as stent thrombosis, which can be a life-threatening consequence of premature discontinuation of DAPT. Furthermore, pooling events for which the intervention has opposing effects (e.g. bleeding versus MI and stent thrombosis) as a composite endpoint may not be appropriate. Future studies are unlikely to be adequately powered for to evaluate the individual bleeding and ischemic endpoints as event rates are now usually low. Therefore, we aimed to perform a study-level meta-analysis of randomized controlled trials (RCTs) to provide further insights on the impact of interrupting DAPT after 1 month following PCI on the individual safety endpoints such stent thrombosis, myocardial infarction (MI), stroke, major bleeding and mortality.

## Methods

This study was performed according to the Cochrane Handbook for Systematic Reviews of Interventions [7] recommendations.

### Eligibility criteria

All RCTs investigating one-month versus routine duration of DAPT in patients undergoing PCI and reporting outcomes from the time of cessation of DAPT (1 month) to 1 year were eligible for inclusion in the meta-analysis. RCTs comparing different stent platforms with fixed duration of DAPT (i.e. one-month DAPT in both arms) were excluded. RCTs only including patients on oral anticoagulation were also excluded.

### Search strategy

We searched PubMed/MEDLINE and Ovid/Embase from inception through to the 17th of September 2021 for studies in English and published as a full-text article. The exact search strategy is available in the online appendix. The abstracts of the recent ESC conference were also searched. Furthermore, the references of the eligible papers were screened to identify any other potential studies.

### Study selection

**Fig. 1:**
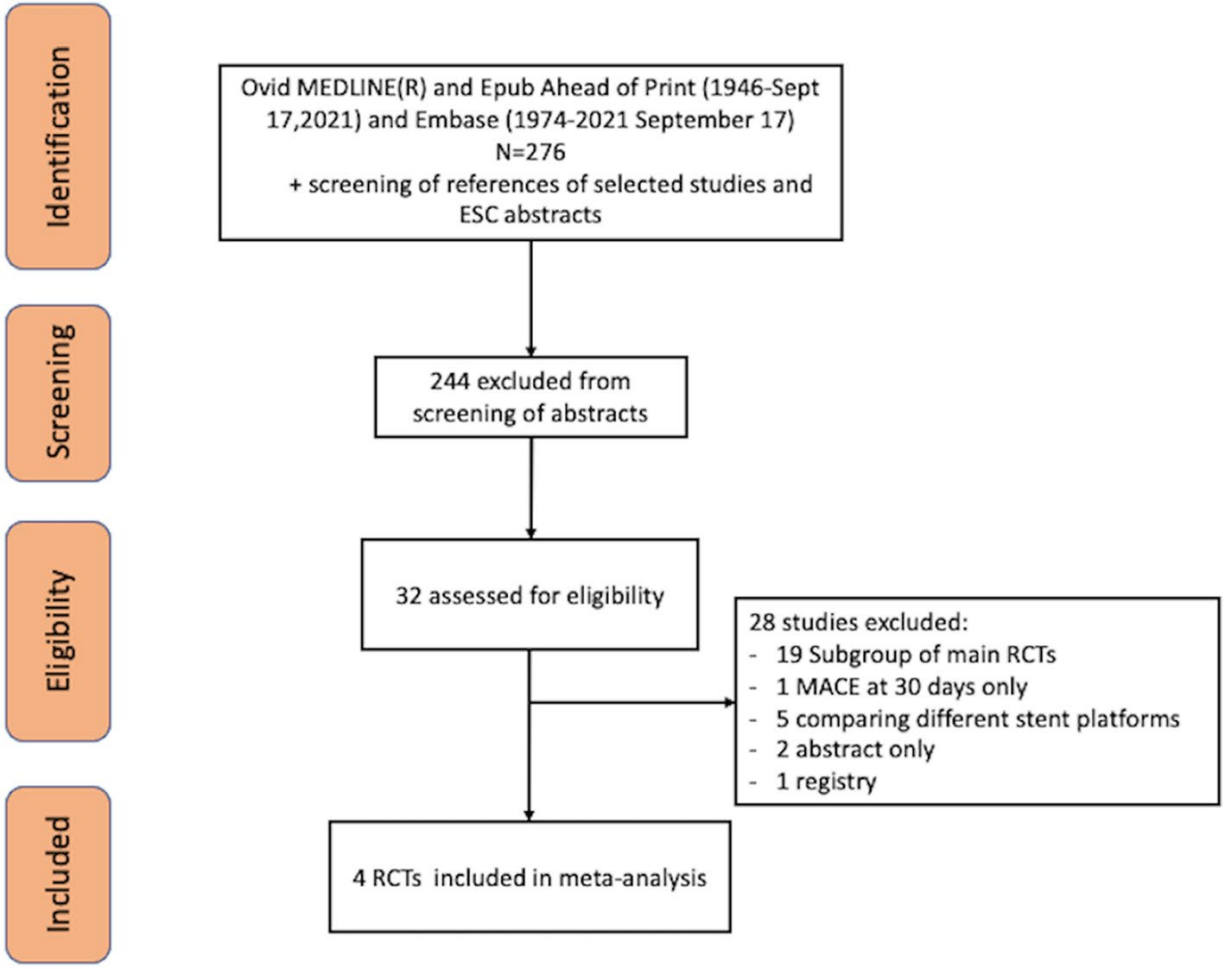
PRISMA flow diagram of the search for published trials showing search strategy with excluded studies and reason for exclusion

Two authors (SP, AA) identified suitable articles and their supplemental appendix and extracted the data independently and in duplication. Any disagreements in data extraction were resolved by a third author (HB). Figure 1 shows the process of study selection as per preferred reporting items for systematic reviews and meta-analyses (PRISMA) [8].

### Data extraction and quality assessment

Characteristics of the RCTs included (study design, intervention performed, number of patients enrolled, inclusion and exclusion criteria, clinical outcomes, and follow-up duration), baseline clinical characteristics of the study population, and individual clinical endpoints were extracted. Risk of bias was assessed as recommended by the Cochrane Handbook [7] (see Online Appendix) but without constructing a composite quality score given the limitations inherent to such an approach [9]. We aimed to produce a funnel plot if there were > 10 included RCTs in the forest plot to assess for publication bias.

### Endpoints

The main safety endpoints of interest analyzed were mortality, MI, stent thrombosis, stroke and major bleeding. Mortality was divided into cardiovascular or non-cardiovascular and stroke as ischemic or hemorrhagic only if these data were available from all selected RCTs. Stent thrombosis was assessed as a combination of definite and probable stent thrombosis when available. Major bleeding was assessed as defined by the Bleeding Academic Research Consortium (BARC) or by the trial definition if BARC classification not available. Outcomes were assessed from 1 month onwards and censored at 1 year for RCTs reporting longer duration of follow-up. Event rates were extracted from the intention-to-treat analysis from each trial.

### Statistical analysis

Statistical analysis was performed using RevMan 5.4 (Nordic Cochrane Centre). The risk of bias assessment of the included RCTs was performed in keeping with the revised Cochrane risk of bias tool (RoB2). Risk ratios (RRs) with 95% confidence intervals (CI) were used as summary estimates. Heterogeneity among trials was quantified using I^2^ statistics with I^2^ of 0–25%, 25–50% and 50–75% considered as low, moderate and high heterogeneity, respectively. The pooled RR was calculated with the random-effects model using the Mantel-Haenszel method. All reported *P* values are two-sided, with significance set at *P* < 0.05. For sensitivity analysis, we tested the robustness of each result by removing one trial at a time.

## Results

### Selection of RCTs

Figure 1 shows the PRISMA diagram of the search and selection strategy. The methodology of the search strategy is available in the online Supplemental Appendix. A total of 276 studies were identified and 32 were eventually selected for full-text review. After reviewing the references of the shortlisted studies and the recent ESC abstracts, 4 RCTs [6, 10–12] met the inclusion criteria to be included in the meta-analysis. One RCTs reported outcomes at 2 years but landmark analyses were available in the online appendix for outcomes from 30 days to 1 year [12]. One RCT randomized patients after 30 days and reported 1 year outcome [6] and the 2 remaining RCTs [10, 11] reported landmark analyses in their online appendices for outcomes between 31 days and 1 year.

### Included studies

The characteristics of the 4 RCTs [6, 10–12] are provided in Table 1. All RCTs were open-label multicentre trials. The GLOBAL LEADERS trial (A Clinical Study Comparing Two Forms of Anti-platelet Therapy After Stent Implantation) [12] recruited 15,968 patients undergoing PCI with the bioabsorbable polymer DES (BP-DES) Biolimus A9 and the participants were randomized to DAPT with aspirin and ticagrelor for 1 month followed by ticagrelor monotherapy for 23 months or DAPT with aspirin and clopidogrel for 12 months. They excluded patients on oral anticoagulation. The MASTER DAPT trial [6] included 4434 participants at high bleeding risk undergoing PCI with a BP-DES (Ultimaster) and they were randomized after 1-month treatment to either cessation of DAPT or to continue DAPT for a minimum of 3 months (median: 193 days). There were 33% of patients on oral anticoagulation in this trial [6]. The STOPDAPT-2 trial (Effect of 1 Month Dual Antiplatelet Therapy Followed by Clopidogrel vs 12-Month Dual Antiplatelet Therapy on Cardiovascular and Bleeding Events in Patients Receiving PCI) [11] randomized 3009 participants from Japan undergoing PCI with the durable polymer-DES (DP-DES) cobalt-chromium everolimus-eluting stent (Xience series) to either one-month DAPT followed by clopidogrel monotherapy or 12 months DAPT with aspirin and clopidogrel. Patients on oral anticoagulation were also excluded from this trial. Finally, the One-month DAPT trial (1-Month Dual-Antiplatelet Therapy Followed by Aspirin Monotherapy After Polymer-Free Drug-Coated Stent Implantation) [10] randomized 3020 South Korean participants to either 1-month DAPT with aspirin and clopidogrel and then aspirin thereafter with PCI with a polymer-free drug coated stent (PF-DCS - Biofreedom) or to 6–12 months DAPT with aspirin and clopidogrel and PCI with BP-DES (Biomatrix and Ultimaster in 99% of cases). Patients on oral anticoagulants were also excluded from this trial. The major inclusion and exclusion criteria of these 4 RCTs are summarised in the Online Table 1 in the Supplemental Appendix.

**Table 1:** Characteristics of RCTs included

Study	Recruitment period and number of participants	Trial design	Stent platform	Experimental and control treatment	Primary endpoints	Findings
**GLOBAL LEADERS**	2013 to 2015 *N* = 15,968 18 countries	Multicentre, open-label, randomized superiority trial	BP-DES (Biolimus A9-eluting stent)	**Experimental arm:** 75–100 mg aspirin daily plus 90 mg ticagrelor twice daily for 1 month, followed by 23 months of ticagrelor monotherapy **Control arm:** standard dual antiplatelet therapy with 75–100 mg aspirin daily plus either 75 mg clopidogrel daily (for patients with stable coronary artery disease) or 90 mg ticagrelor twice daily (for patients with acute coronary syndromes) for 12 months, followed by aspirin monotherapy for 12 months	A composite of all-cause mortality or non-fatal centrally adjudicated new Q-wave MI at 2 years	Ticagrelor in combination with aspirin for 1 month followed by ticagrelor alone for 23 months was not superior to 12 months of standard dual antiplatelet therapy followed by 12 months of aspirin alone in the prevention of all-cause mortality or new Q-wave MI 2 years after PCI
**MASTER DAPT**	2017 to 2019 *N* = 4434 30 countries	Multicentre, randomized, open-label, noninferiority trial with sequential superiority testing	BP-DES (Ultimaster, Terumo)	**Experimental arm:** discontinuation of DAPT after one month Median duration of DAPT: 34 days **Control arm:** Continue DAPT for at least 2 additional months Median duration of DAPT: 193 days	Net adverse clinical events (a composite of death from any cause, MI, stroke, or major bleeding) Major adverse cardiac or cerebral events (a composite of death from any cause, MI, or stroke) Major or clinically relevant nonmajor bleeding at 1 year	One month of DAPT was noninferior to the continuation of therapy for at least 2 additional months; abbreviated therapy also resulted in a lower incidence of major or clinically relevant non-major bleeding.
**STOPDAPT-2**	2015 to 2017 *N* = 3009 Japan	Multicentre, open-label, adjudicator-blinded randomized controlled trial	DP-DES (Cobalt-chromium everolimus-eluting stent Xience Series, Abbott Vascular)	**Experimental arm:** 1-month DAPT followed by clopidogrel monotherapy **Control arm:** 12 months of DAPT with aspirin and clopidogrel	Composite of cardiovascular death, MI, ischemic or hemorrhagic stroke, definite stent thrombosis or major or minor bleeding at 12 months.	1-month DAPT followed by clopidogrel monotherapy compared with 12 months of DAPT with aspirin and clopidogrel resulted in a significantly lower rate of a composite of cardiovascular and bleeding events
**One-month DAPT**	2015 to 2019 *N* = 3020 South Korea	Multicentre, randomized, open-label trial	**Experimental arm:** PF-DCS (Biofreedom) **Control arm:** BP-DES (predominantly Biomatrix and Ultimaster in 99% of cases)	**Experimental arm:** 1-month DAPT with aspirin and clopidogrel, then aspirin thereafter Median duration of DAPT: 1.1 months **Control arm:** 6–12 months of DAPT with aspirin and clopidogrel Median duration of DAPT: 12 months	1 year composite of cardiac death, non-fatal MI, target vessel revascularisation, stroke, or major bleeding	1-month DAPT followed by aspirin after PF-DCS was non-inferior to 6–12 months of DAPT after BP-DES for the 1-year composite endpoint.

*BP-DES* biodegradable-polymer drug eluting stent, *DAPT* dual antiplatelet therapy, *DP-DES* durable-polymer drug eluting stent, *PF-DCS* polymer-free drug coated stent, *MI* myocardial infarction

Therefore a total of 26,576 patients from these 4 RCTs [6, 10–12] were included in the pooled analysis. The mean age of the participants ranged between 65 to 76 years old and the percentage of women ranged between 22 to 31%. Stable angina ranged between 40 to 62%. Further details of the baseline patient characteristics and clinical presentation are provided in Table 2.

**Table 2:** Baseline patient demographics

Study	Mean Age	Female	Diabetes Mellitus	Hypertension	Dyslipidemia	Current Smoker	Previous PCI	Need for oral anticoagulation	Clinical presentation	Non-adherence rate
**GLOBAL LEADERS**	65	23%	25%	74%	70%	26%	33%	Exclusion criteria	Stable angina: 53% NSTEMI: 21% STEMI: 13%	Experimental arm: 27% Control arm: 13%
**MASTER DAPT**	76	31%	33%	77%	68%	9%	26%	33%	Stable angina: 40% NSTEMI: 25% STEMI: 12%	Experimental arm: 2% Control arm: 0.4%
**SHORTDAPT-2**	69	22%	39%	74%	75%	24%	34%	Exclusion criteria	Stable angina: 62% NSTEMI: 6% STEMI: 19%	Experimental arm: 0.1% Control arm: None
**One-month DAPT**	67	31%	38%	67%	82%	17%	17%	Exclusion criteria	Stable angina: 62% Unstable angina: 37%	Experimental arm: 18% Control arm: 4%

*PCI* percutaneous coronary intervention, *NSTEMI* non-ST segment elevation myocardial infarction, *STEMI* ST-segment elevation myocardial infarction

### Risk of bias assessment

The risk of bias assessment is detailed in the Online Table 2 in the Supplemental Appendix. MASTER DAPT [6] and SHORTDAPT-2 [11] trials were at low risk of bias. However, there were some concern for risk of bias for GLOBAL LEADERS [12] and One-month DAPT [10] trials. In GLOBAL LEADERS [12], this was predominantly due to an imbalance in the non-adherence rate of the allocated intervention (27% non-adherence in the experimental arm and 13% in the control arm) and this was an open-label RCT with the event rates in the majority of cases not independently adjudicated. In One-month DAPT [10], the non-adherence rate was also imbalanced between the 2 arms (18% in the intervention arm versus 4% in the control arm) and the stent platforms used in each arm were also different.

### Clinical efficacy and safety outcomes

The event rates below were between 1 month and 12 months, after cessation of DAPT in the experimental arm. The duration of DAPT in the control arm ranged between 3 to 12 months.

#### Major bleeding

**Fig. 2:**
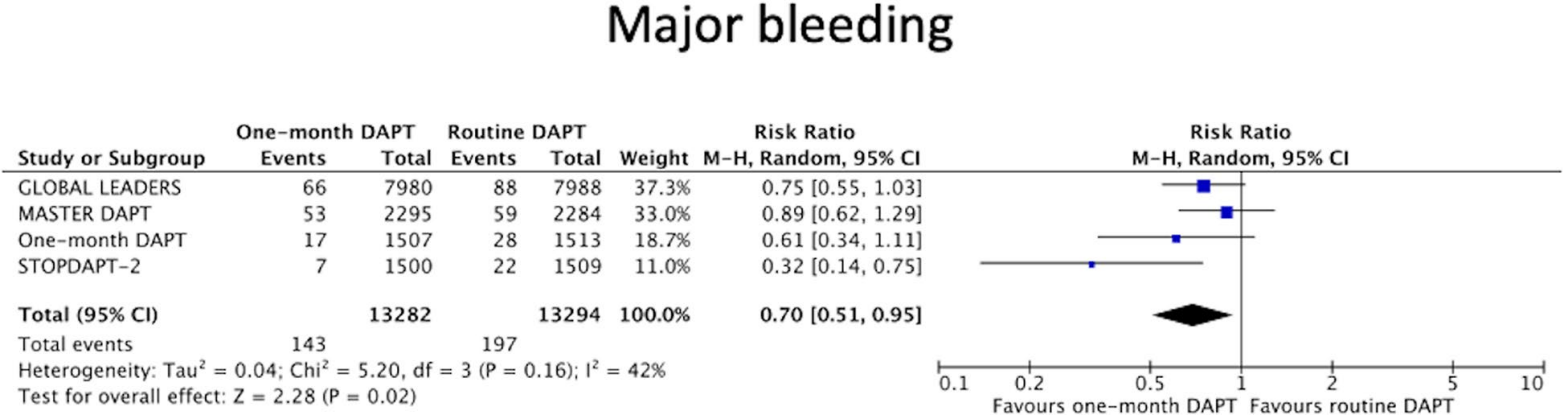
Forest plots comparing one-month DAPT to routine DAPT for major bleeding

Three RCTs [6, 11, 12] defined major bleeding by Bleeding Academic Consortium (BARC) criteria and 1 RCT [10] used the Safety and Efficacy of Enoxaparin in PCI Patients, an international Randomized Evaluation (STEEPLE) definition. Cessation of DAPT after 1 month was associated with significantly less major bleeding when compared to the control arm [1.1% versus 1.5%, weighted absolute risk difference 0.49 (95%CI 0.11–0.87)%, *P* = 0.05, Random-effects model RR 0.70, 95%CI (0.51–0.95), *P* = 0.02, I^2^ = 42%, Fig. 2].

#### Mortality

**Fig. 3:**
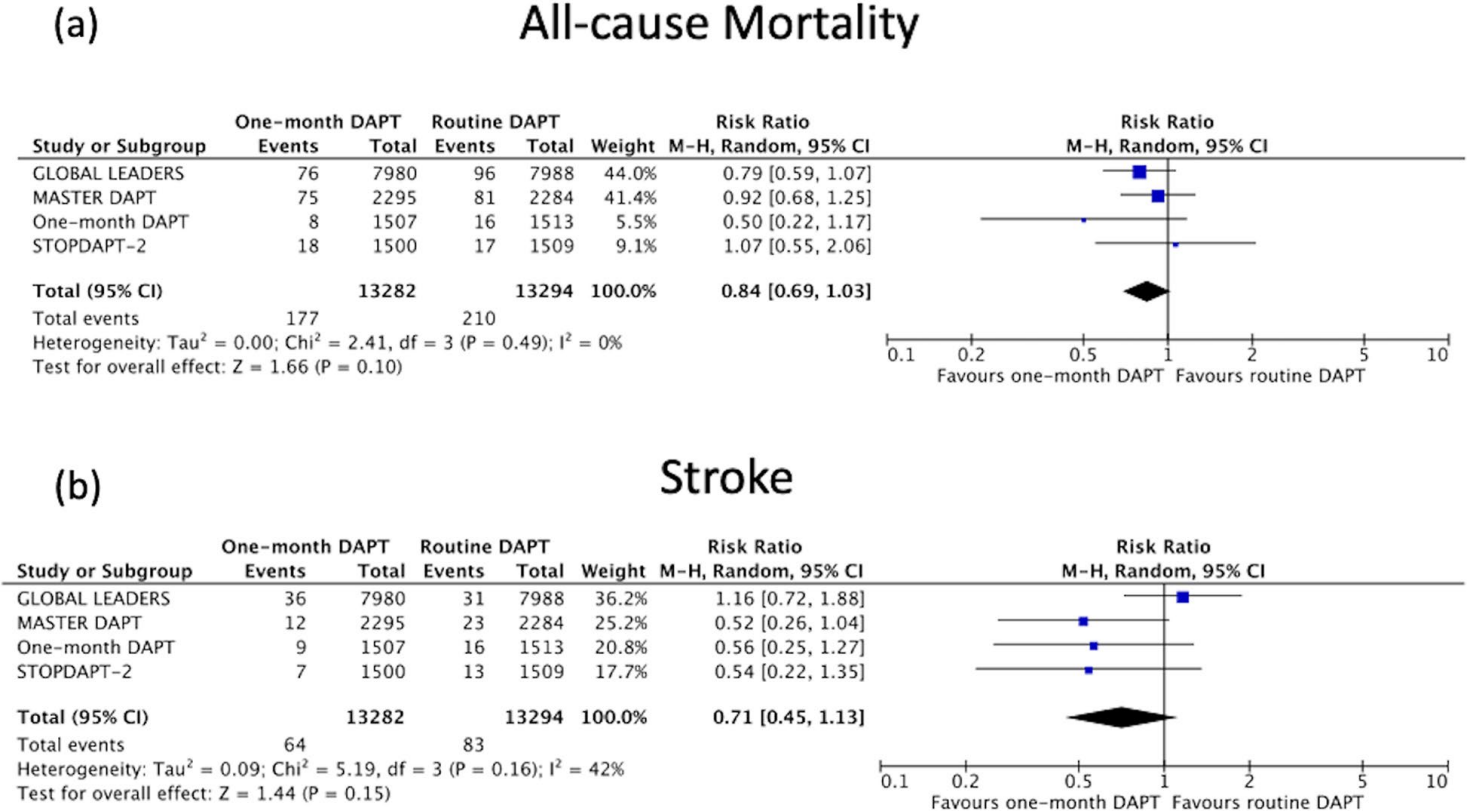
Forest plot comparing one-month DAPT to routine DAPT for (**a**) all-cause mortality and (**b**) stroke

There was no significant difference in all-cause mortality with cessation of DAPT after 1 month when compared to the control arm [1.3% versus 1.6%, weighted absolute risk difference 0.26 (95%CI 0.00–0.52)%, *P* = 0.05, Random-effects model RR 0.84 (95%CI 0.69–1.03), *P* = 0.10, I^2^ = 0%, Fig. 3a].

Cardiovascular mortality was only reported in 2 RCTs [6, 11] with a further RCT [10] reporting cardiac mortality only. There was no difference in cardiovascular mortality when data from the 2 RCTs were pooled [1.1% versus 1.4%, Random-effects model RR 0.80 (95%CI 0.53–1.18), *P* = 0.56, I^2^ = 0%].

#### Stroke

The stroke endpoint was a combination of ischemic and hemorrhagic stroke as not all RCTs reported these individual components separately. There was no statistically significant difference in stroke with cessation of DAPT after 1 month when compared to the control arm [0.5% versus 0.6%, weighted absolute risk difference 0.24 (95%CI -0.10-0.58)%, *P* = 0.16, Random-effects model RR 0.71 (95%CI 0.45–1.13), *P* = 0.15, I^2^ = 42%, Fig. 3b].

#### Myocardial infarction and stent thrombosis

**Fig. 4:**
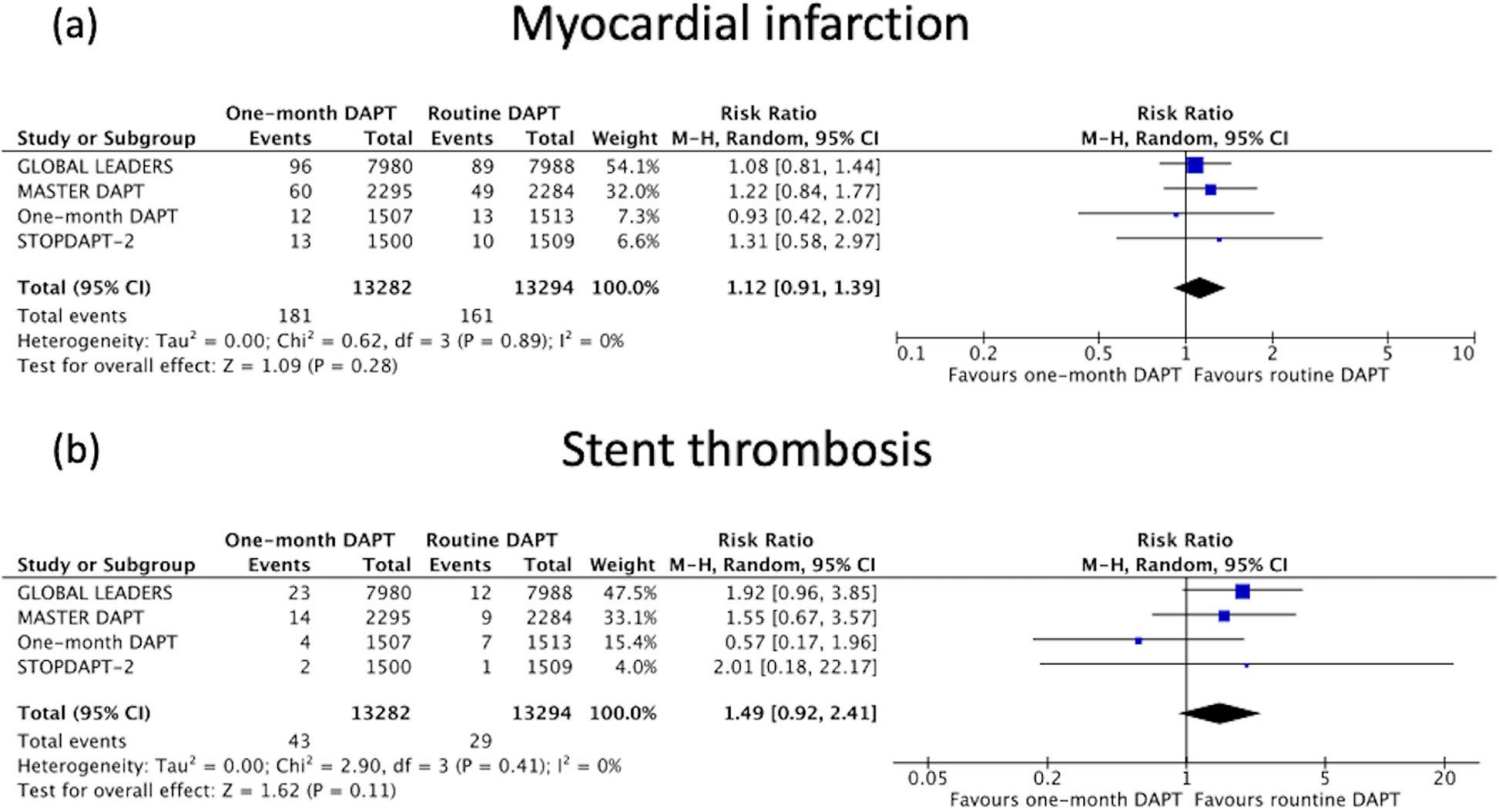
Forest plot comparing one-month DAPT to routine DAPT for (**a**) myocardial infarction and (**b**) stent thrombosis

There was also no statistically significant difference in MI [1.4% versus 1.2%, weighted absolute risk difference − 0.11 (95%CI -0.37-0.14)%, *P* = 0.37, Random-effects model RR 1.12 (95%CI 0.91–1.39), *P* = 0.28, I^2^ = 0%, Fig. 4a], and definite or probable stent thrombosis [0.3% versus 0.2%, weighted absolute risk difference − 0.09 (95%CI -0.23-0.05)%, *P* = 0.19, Random-effects model RR 1.49 (95%CI 0.92–2.41), *P* = 0.11, I^2^ = 0%, Fig. 4b], with cessation of DAPT after 1 month when compared to the control arm.

### Sensitivity analysis

The results for all-cause mortality and MI did not differ when one trial at a time was removed from the analysis. However, cessation of DAPT after 1 month was associated with significantly more stent thrombosis [Random-effects model RR 1.77 (95%CI 1.05–2.98), *P* = 0.03, I^2^ = 0%] and the difference in major bleeding was no longer statistically significant [Random-effects model RR 0.70 (95%CI 0.47–1.05), *P* = 0.08, I^2^ = 58%] when One-month DAPT trial [10] was removed from the analysis. There was also significantly less stroke after 1 month DAPT cessation [Random-effects model RR 0.54 (95%CI 0.34–0.85), *P* = 0.008, I^2^ = 0%] when GLOBAL LEADERS [12] was removed from the analysis.

## Discussion

Our meta-analysis of 4 RCTs showed that cessation of DAPT after 1 month following PCI was associated with significantly less bleeding and there was no difference in all-cause mortality, stroke, MI and stent thrombosis up to 1 year follow-up, when compared to 3–12 months DAPT duration.

These data suggest that shorter DAPT reduces bleeding risk, but this may be at a cost of an increase in ischemic events due to the wide 95% confidence intervals (stent thrombosis: ranging between a potential relative reduction of 9% to a relative increase of 41%; MI: potential relative reduction of 9% to relative increase of 39%), although the differences were not statistically significant. Furthermore, there was a trend towards less 1-year mortality, as highlighted by the wide 95% confidence intervals, in the 1-month DAPT group (all-cause mortality: ranging between a potential relative reduction of 31% to a relative increase of 3%; cardiovascular mortality: potential relative reduction of 47% to relative increase of 18%). The trend in reduction in all-cause mortality may be due to less bleeding-related deaths in the 1-month DAPT group and the trend in a reduction in cardiovascular mortality may be due to a less stroke-related (likely haemorrhagic) deaths, as there was a trend towards less stroke in the 1-month DAPT group. The other explanation could be that the trends seen could be due by chance as the event rates for all-cause mortality was very low (1.3 and 1.6% in each arm for the follow-up period between 1 month and 12 months) and to be adequately powered to detect such a difference, a sample size of > 50,000 patients would be required. Such a megatrial is unlikely to happen and our analysis provides some insights of risk benefits of 1-month DAPT and the event rates provided could be used to inform physicians and patients involved in the decision making.

Of note, although the MASTER DAPT trial recruited HBR patients, the criteria to qualify as HBR risk was relatively broad and only around half of trial population had a PRECISE-DAPT score of > 25 (definition for high risk for bleeding) [6]. The SHORTDAPT-2 ACS trial [13] was recently presented at the ESC congress 2021. This trial included a subset of ACS patients from the SHORTDAPT 2 trial included in this meta-analysis and has not yet been published in full-text format. It showed that 1-month DAPT followed by clopidogrel monotherapy for 11 months did not meet criteria for noninferiority compared with 12-month DAPT for the composite ischemic/bleeding endpoint among ACS patients undergoing PCI with a DP-DES (Xience series). There was a trend towards harm for the composite ischemic endpoint in the 1-month DAPT arm, with a significant nearly 2-fold increase in the risk of MI. SHORTDAPT 2 results are concordant with our data, in which approximately half of the participants presented with an ACS, with a trend towards increased MI in our meta-analysis.

However, no RCTs so far have recruited HBR patients with a PRECISE-DAPT score of > 25 only or those with ACS undergoing PCI with a PF-DCS or BP-DES. Furthermore, the overall rate of stent thrombosis between 1 month and 1 year was 0.3% in the one-month DAPT arm compared to 0.2% in the routine DAPT (3–12 months DAPT) arm. Future trials looking at these individual endpoints may not be feasible as they would require a very large sample size. Therefore, a pragmatic approach based on the evidence so far may be: one-month DAPT should be considered in HBR patients such as a PRECISE DAPT score of > 25. For those undergoing complex PCI (e.g. LMS bifurcation stenting, long segments of overlapping stents etc), an individualised approach should be adopted. Whether using the latest generation BP-DES with thinner struts and intravascular imaging-guided PCI may provide more confidence to stop DAPT after 1 month in some patients needs to be investigated in future trials.

To our knowledge, this is the first meta-analysis of more than 25,000 patients and looking at clinical outcomes after cessation of DAPT, from 1 month to 1 year. However, our meta-analysis has several limitations. This was study-level rather than patient-level meta-analysis and therefore we could not stratify patients by clinical presentation, choice of stent, choice of antiplatelet therapy after DAPT cessation and HBR category. Only one trial included a minority of patients with concomitant oral anticoagulation and therefore these findings do not apply to those patients. Of note, a previous meta-analysis showed that in patients with atrial fibrillation undergoing PCI, treatment with an oral anticoagulation and one antiplatelet agent only was associated with significantly less bleeding but no difference in ischemic events when compared to triple therapy [14]. The trend in a reduction in stroke may have been due to a reduction in hemorrhagic stroke but unfortunately the breakdown in the cause of death or type of strokes were not available in all of the included RCTs. Bias assessment showed that there were some concern with two of the included RCTs [10, 12] and this may explain some of the findings of the sensitivity analysis. The portion of patients with chronic coronary syndrome varied among the included RCTs and so did the duration of DAPT in the control arm and the follow-up duration. Lastly, cardiac death was only provided from 1 RCT and therefore we could not comment on whether the trend in more MI and stent thrombosis translated to a signal of more cardiac deaths in the RCTs included in our analysis. Cardiac mortality rather than cardiovascular or all-cause mortality would be more ideal when evaluating the impact of MI or stent thrombosis, but this endpoint is more challenging to adjudicate and most studies report all-cause mortality, which is more pertinent as a patient-oriented end point.

## Conclusion

Among RCTs evaluating one-month DAPT versus routine DAPT in patients undergoing PCI with predominantly BP-DES or BF-DES, cessation of DAPT after 1 month was associated with significantly less major bleeding. There was no significant difference in the rate of all-cause mortality, stroke, MI and stent thrombosis during the follow-up period of 1 month to 1 year.

## Supplementary Information

**Additional file 1: **Search strategy. **Online Table 1.** Inclusion and exclusion criteria. **Online Table 2.** Risk of bias assessment. **Online Table 3.** Event rates from one month to 1 year.

## Data Availability

The data and materials related to this meta-analysis are available upon request from the corresponding author.

## Abbreviations

DAPT: Dual antiplatelet therapy
PCI: Percutaneous coronary intervention
DES: Drug-eluting stent
HBR: High bleeding risk
ESC: European society of cardiology
RCTs: Randomized controlled trials
MI: Myocardial infarction
PRISMA: Preferred reporting items for systematic reviews and meta-analyses
BARC: Bleeding academic research consortium
RR: Risk ratio
CI: Confidence interval
BP: Bioabsorbable polymer
