# Supplementary material for: Safety and efficacy of interrupting dual antiplatelet therapy one month following percutaneous coronary intervention: a meta-analysis of randomized controlled trials
Source: BMC Cardiovasc Disord. 2022 Oct 28;22:450. doi: 10.1186/s12872-022-02900-6 (PMC9615356; doi:10.1186/s12872-022-02900-6)
Supplement: Supplementary file 1 — Additional file 1: Search strategy. Online Table 1. Inclusion and exclusion criteria. Online Table 2. Risk of bias assessment. Online Table 3. Event rates from one month to 1 year. [file 12872_2022_2900_MOESM1_ESM.docx]

Supplemental Appendix

Table of Contents

[Search strategy 2](#_Toc83045123)

[Online Table 1: Inclusion and exclusion criteria 3](#_Toc83045124)

[Online Table 2: Risk of bias assessment 6](#_Toc83045125)

[Online Table 3: Event rates from one month to 1 year 7](#_Toc83045126)

# Search strategy

Database: Embase <1974 to 2021 September 17>, Ovid MEDLINE(R) ALL <1946 to September 17, 2021>

Search Strategy:

--------------------------------------------------------------------------------

1 dual antiplatelet therapy.mp. [mp=ti, ab, hw, tn, ot, dm, mf, dv, kf, fx, dq, nm, ox, px, rx, an, ui, sy] (20089)

2 duration.mp. [mp=ti, ab, hw, tn, ot, dm, mf, dv, kf, fx, dq, nm, ox, px, rx, an, ui, sy] (1960822)

3 1 and 2 (3733)

4 percutaneous coronary intervention.mp. [mp=ti, ab, hw, tn, ot, dm, mf, dv, kf, fx, dq, nm, ox, px, rx, an, ui, sy] (138433)

5 3 and 4 (1838)

6 short.mp. [mp=ti, ab, hw, tn, ot, dm, mf, dv, kf, fx, dq, nm, ox, px, rx, an, ui, sy] (2590284)

7 5 and 6 (390)

8 remove duplicates from 7 (276)

***************************

# Online Table 1: Inclusion and exclusion criteria

| **Study** | **Inclusion criteria** | **Exclusion criteria** |
| --- | --- | --- |
| **GLOBAL LEADERS** | Age **≥**18 years  Presence of one or more coronary artery stenoses of 50% or more in a native  coronary artery or in a saphenous venous or arterial bypass conduit suitable  for coronary stent implantation  Able to provide informed consent and willing to participate in 2 year follow-up  period. | Known intolerance to aspirin, P2Y12 inhibitors, bivalirudin, stainless steel or biolimus  Known intake of a strong CYP3A4 inhibitor (e.g., ketoconazole,  clarithromycin, nefazodone, ritonavir, and atazanavir), as co-administration may lead to a substantial increase in exposure to ticagrelor  Known moderate to severe hepatic impairment (alanine-aminotransferase **≥**3 x ULN)  Planned surgery, including CABG as a staged procedure (hybrid) within 12 months of the index procedure, unless dual antiplatelet therapy is maintained throughout the peri-surgical period  Need for chronic oral anti-coagulation therapy  Active major bleeding or major surgery within the last 30 days  Known history of intracranial haemorrhagic stroke or intra-cranial aneurysm  Known stroke (any type) within the last 30 days  Known pregnancy at time of randomisation  Female who is breastfeeding at time of randomisation  Currently participating in another trial and not yet at its primary endpoint |
| **MASTER DAPT** | Age≥18 years  At least 1 HBR criterion  All coronary lesions are successfully treated with Ultimaster (TANSEI) stent  Free of any flow-limiting angiographic complications which required prolonged DAPT duration based on operator's decision  All stages of PCI are complete (if any), and no further PCI is planned  Inclusion criteria at 1-m randomization visit (30-44 d after qualifying index PCI)  At least 1 HBR criterion (listed above) or on the basis of post-PCI actionable non–access-site related bleeding episode  Uneventful 30-d clinical course (ie, new episode of acute coronary syndrome, symptomatic restenosis, ST, stroke, any revascularization requiringprolonged DAPT)  If not on OAC:a) Patient is on DAPT regimen of aspirin and a P2Y12 inhibitor;b) Patient with 1 type of P2Y12 inhibitor for at least 7 d  If on OAC:a) Patient is on the same type of OAC for at least 7 d;b) Patient is on clopidogrel for at least 7 d | Treated with stent other than Ultimaster (TANSEI) stent within 6 m prior to index PCI  Treated for in-stent restenosis or stent thrombosis at index PCI or within 6 m before  Treated with a bioresorbable scaffold at any time prior to index procedure  Incapable of providing written informed consent  Under judicial protection, tutorship, or curatorship  Unable to understand and follow study-related instructions or unable to comply with study protocol  Active bleeding requiring medical attention (BARC≥2) on randomization visit  Life expectancy less than 1 y  Known hypersensitivity or allergy for aspirin, clopidogrel, ticagrelor, prasugrel, cobalt-chromium, or sirolimus  Any planned and anticipated PCI  Participation in another trial  Pregnant or breastfeeding women |
| **STOPDAPT-2** | Patients who underwent successful PCI with CoCr-EES (Xience Series, Abbott Vascular) without concomitant use of other types of drug-eluting stent or in-hospital major complications other than periprocedural MI | Need for oral anticoagulation or antiplatelet therapy other than aspirin and P2Y12 receptor blockers, history of intracranial bleeding, and known intolerance to clopidogrel |
| **One-month DAPT** | Patients ≥19 years old  Patients with ischemic heart disease who are considered for coronary revascularization with stent implantation.  Significant coronary de novo lesion | Acute myocardial infarction  Complex lesion morphologies such as aorto-ostial, unprotected left main, chronic total occlusion, graft, thrombosis, heavy calcified or extremely tortuous lesion  Need to use of DAPT more than 1 month because of other medical conditions  Cardiogenic shock or experience of cardiopulmonary resuscitation  Contraindication or hypersensitivity to Biolimus A9 or Sirolimus, stainless steel, heparin, antiplatelet agents or contrast media  History of documented prior cerebrovascular attack within 6 months  Treated with any stent within 3 months  Reference vessel diameter <2.25 mm or >4.0 mm  Pregnant women or women with potential childbearing  Inability to follow the patient over the period of 1 year after enrollment, as assessed by the investigator  Inability to understand or read the informed content |

# Online Table 2: Risk of bias assessment

| **Study** | **Risk of bias arising from the randomization process** | **Risk of bias due to deviations from the intended interventions** | **Missing outcome data** | **Risk of bias in measurement of the outcome** | **Risk of bias in selection of the reported result** | **Overall risk of bias** |
| --- | --- | --- | --- | --- | --- | --- |
| **GLOBAL LEADERS** | Low | Some concern | Low | Some concern | Low | Some concern |
| **MASTER DAPT** | Low | Low | Low | Low | Low | Low |
| **STOPDAPT-2** | Low | Low | Low | Low | Low | Low |
| **One-month DAPT** | Low | Some concern | Low | Low | Low | Some concern |

# Online Table 3: Event rates from one month to 1 year

|  | **GLOBAL LEADERS** | | **MASTER DAPT** | | **STOPDAPT-2** | | **One-month DAPT** | |
| --- | --- | --- | --- | --- | --- | --- | --- | --- |
|  | Experimental  N=7980 | Control  N=7988 | Experimental  N=2295 | Control  N=2284 | Experimental  N=1500 | Control  N=1509 | Experimental  N=1507 | Control  N=1513 |
| All-cause mortality | 76 | 96 | 75 | 81 | 18 | 17 | 8 | 16 |
| Cardiovascular mortality | NA | NA | 37 | 44 | 6 | 10 | 2* | 6* |
| Myocardial infarction | 96 | 89 | 60 | 49 | 13 | 10 | 12 | 13 |
| Stroke | 36 | 31 | 12 | 23 | 7 | 13 | 9 | 16 |
| Revascularisation | 406 | 407 | NA | NA | 97 | 73 | 37 | 34 |
| Definite stent thrombosis | 23 | 12 | 11 | 7 | 2 | 1 | 4 | 7 |
| Probable stent thrombosis | NA | NA | 3 | 2 | 0 | 0 |  |  |
| Major Bleeding | 66  BARC 3 or 5 | 88  BARC 3 or 5 | 53  BARC 3 or 5 | 59  BARC 3 or 5 | 7  BARC 3 or 5 | 22  BARC 3 or 5 | 17  STEEPLE | 28  STEEPLE |

*cardiac death rather than cardiovascular death
